# Pyroptosis-Related Gene Signature Predicts the Prognosis of ccRCC Using TCGA and Single-Cell RNA Seq Database

**DOI:** 10.1155/2022/8224618

**Published:** 2022-10-30

**Authors:** Ying Gan, Zhenan Zhang, Xiaofei Wang, Aolin Li, Yu Fan, Qian Zhang

**Affiliations:** Department of Urology, Peking University First Hospital, Beijing 100034, China

## Abstract

Clear cell renal cell carcinoma (ccRCC) is the most prevalent type of renal carcinoma, which is not sensitive to both radiotherapy and chemotherapy. The objective response rate of metastatic renal cancer to targeted drugs and immunotherapy is unsatisfactory. Pyroptosis, proven as an inflammatory form of programmed cell death, could be activated by some inflammasomes, while could create a tumor-suppressing environment by releasing inflammatory factors in the tumor. To explore indicators predicting the prognosis of ccRCC and the effect of antitumor therapy, we constructed a pyroptosis risk model containing 4 genes after 11 pyroptosis-related genes of 516 ccRCC cases in the TCGA database were scanned. Based on the risk score, 516 ccRCC cases were divided into two groups for functional enrichment analysis and immune profile to seek functional pathways and potential therapeutic targets. Besides, those results were verified in GSE29609 and single-cell transcriptomic data. The study suggests that the conducted pyroptosis model could predict the prognosis of ccRCC and reflect the immune microenvironment, which may help in immune checkpoint inhibitor treatment.

## 1. Introduction

Renal cell carcinoma (RCC), which originates from renal proximal convoluted tubule epithelial cells, accounts for about 90% of all primary renal malignancies. RCC is one of the most prevalent malignant tumors of the genitourinary system. In recent decades, the incidence of RCC continues to rise [1]. According to histological classification, clear cell renal cell carcinoma (ccRCC) is the most common type of renal carcinoma (about 75–80%), followed by papillary carcinoma (15%) and chromophobe cell carcinoma (5%) [2]. The onset of RCC is insidious and lacks specific clinical manifestations and features at the early stage. About 20–30% of RCC has been found with metastasis at the time of initial diagnosis [3]. RCC is not sensitive to both radiotherapy and chemotherapy, which currently mainly relied on surgical resection. Despite the development of targeted drugs and immunotherapy in recent years, the objective response rate of metastatic renal cancer is only about 30% [4]. Although some clinical indicators and pathological results have been used to predict the treatment and prognosis of ccRCC, their predictive ability is insufficient [5]. Consequently, it does make sense to explore indicators predicting the prognosis of ccRCC.

Pyroptosis is an inflammatory form of programmed cell death. It is triggered by caspase-1/4/5/11, which is activated by some inflammasomes. Pyroptosis causes cell swelling, dissolution of the plasma membrane, fragmentation of chromatin, and freeing of inflammatory content of intracellular proteins [6]. Gasdermin superfamilies are the main effectors of pyroptosis. Gasdermin superfamily members discovered so far include GSDMA, GSDMB, GSMDC, GSDMD, GSDME, DFNB59, and so on [7]. The cleavage of the gasdermin by caspases is the key to activating gasdermin to form permeable pores, but not all gasdermin could be cut by caspases [8, 9]. The endurance to cell death is one of the six hallmarks of cancer. Cell death particularly breaks down into necrosis and programmed cell death, and the latter includes apoptosis, pyroptosis, and autophagy [10]. Promoting pyroptosis of cancer cells could validly inhibit tumorigenesis and tumor progression. It can also raise the effect of antitumor therapy [11]. It has been reported that pyroptosis could create a tumor-suppressing environment with released inflammatory factors in different tumor types. However, pyroptosis can also debilitate the own immune response to cancer cells and quicken tumor growth [12–15].

ccRCC with metastasis is usually incurable by surgical resection and requires systemic treatment [16]. However, metastatic RCC shows insensibility to radiotherapy and systematic treatment in the later stages of treatment, including hormone therapy, chemotherapy, and interleukin-2-based immunotherapy [17]. The study of the CancerGenome Atlas has significantly advanced the molecular classification of renal cell carcinoma to guide the treatment and prognosis. Among them, the activation of protein kinase B (PKB/Akt), the mammalian target of the rapamycin (mTOR) pathway is a key driver of RCC. The expression and activity of mTOR downstream effectors in RCC are unbalanced, which lays a theoretical foundation for the clinical application of ccRCC-targeted therapy [18]. In the last few years, with the in-depth study of cytotoxic T cell inhibitory molecules such as cytotoxic T lymphocyte-associated protein 4, programmed cell death receptor 1 (PD-1), and programmed cell death ligand-1 (PD-L1), the immunomodulators have been applied in clinical practice. Although these treatments improved the prognosis of ccRCC, drug resistance and recurrence still occurred [19, 20]. Due to the lack of a well-established subgroup classification of ccRCC, there is still a lack of molecular subtypes to guide clinical practice. Consequently, it is urgent to construct an effective genetic signature to guide subgroup classification and predict prognosis.

**Figure 1:**
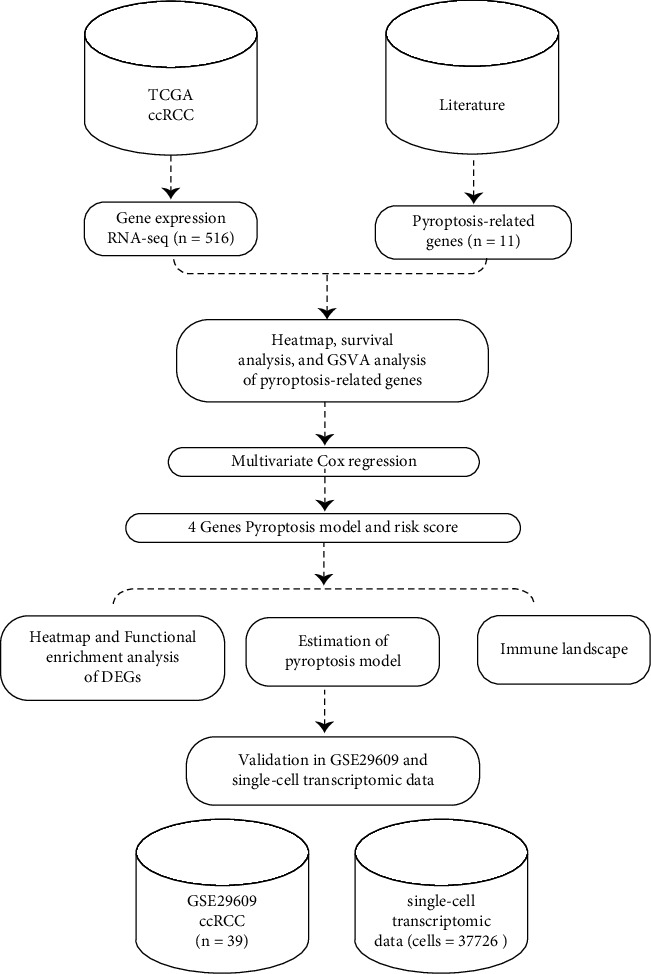
The workflow of the study.

In view of the important part of pyroptosis in the development and treatment of ccRCC, we constructed a pyroptosis risk model to classify ccRCC in the present study to predict prognosis and treatment. 11 pyroptosis-related genes were selected, and survival analysis and GSVA analysis were performed on 516 ccRCC cases in TCGA database. The pyroptosis risk model containing 4 genes was constructed by the multivariate COX regression analysis. Using the risk score, 516 ccRCC cases were grouped into two groups for functional enrichment analysis and immune profile. Those results were verified in GSE29609 and single-cell transcriptomic data. Our findings suggest that the pyroptosis model could predict the prognosis of ccRCC and reflect the immune microenvironment.  Figure 1 shows the flowchart of the study.

## 2. Materials and Methods

### 2.1. Database

The patients' characteristics and renal cell carcinoma patients with level 3 gene expression profiles were downloaded from the TCGA database (June 2020) (https://cancergenome.nih.gov) [21]. We selected the 516 cancer cases whose pathological diagnosis is clear cell renal cell carcinoma (ccRCC). Cases without pathological or clinical information were excluded. GSE29609 dataset [22], as a validation cohort, was obtained from the Gene Expression Omnibus (GEO) database (https://www.ncbi.nlm.nih.gov/geo). The limma package in R was used to normalize the gene expression.

Single-cell transcriptome profiling data for analyses were downloaded from the supplemental data in the published article [23]. The Seurat package in R (version 4.0.4) was applied to process the single-cell RNA-seq data. Cell clusters were recognized by Uniform Manifold Approximation and Projection (UMAP) with a resolution of 0.5 [24]. The function FeaturePlot and VlnPlot of the Seurat package were used for visualization of the expression profiling of the genes.

### 2.2. Identification of Pyroptosis-Related Genes

We selected 11 genes as crucial pyroptosis-related genes which were proved by reliable literature published in the past. The 11 identified genes including CASP1 [25], CASP3 [26], CASP4 [27], CASP5 [28], CASP8 [29], GSDMB [30], GSDMC [31], GSDMD [27], GSDME [26], GZMA [30], and GZMB [32].

### 2.3. Construction of a Risk Model

The heatmaps of pyroptosis-related genes were generated by the pheatmap package in R. Pyroptosis pathway enrichment was performed based on the pyroptosis-related signatures and Gene Set Variation Analysis (GSVA) [33]. R package survival was used for the Kaplan–Meier survival analysis of these 11 pyroptosis-related genes. The function coxPH of the survival package was utilized to generate the Cox proportional hazards regression model. We selected the prognosis-related genes and formed the formula: risk score = *β*1gene1 × expression of gene1 + *β*2gene2 × expression of gene2 +…+ *β*ngenen × expression of genen. The R package survival and ROCR were applied to form the Kaplan–Meier analysis and the receiver operating characteristic (ROC) curves. The predictive value of the new risk model was validated using the GSE29609 dataset downloaded from the GEO database.

### 2.4. Identification of Differentially Expressed Genes and Functional Enrichment Analysis

Cancer cases were grouped into two groups, the high-risk group, and the low-risk group, following the median value of the risk score calculated. The differentially expressed genes (DEGs) between the two groups were identified using the limma package in R with the fold change (|fold change| ≥ 1.5) and adj. *P* < 0.05. The functional enrichment analysis and KEGG (Kyoto Encyclopedia of Genes and Genomes) pathway analysis were conducted using the clusterProfiler package in R.

### 2.5. Assessment of Immune Cell Type Fractions

The analytical web server tool CIBERSORT (https://cibersort.stanford.edu/) was applied to estimate the immunologic cell abundances in the cancer immune microenvironment [34]. The leukocyte gene signature matrix termed LM22 was used to distinguish the 22 immune cell types between the high-and low-risk score groups. The 22 immune cell types including CD8 T cells, naive CD4 T cells, resting memory CD4 T cells, activated memory CD4 T cells, naive B cells, memory B cells, plasma cells, follicular helper T cells, T cells regulatory (Tregs), gamma delta T cells, resting NK cells, activated NK cells, monocytes, macrophages M0, macrophages M1, macrophages M2, resting dendritic cells, activated dendritic cells, resting mast cells, activated mast cells, eosinophils, and neutrophils.

### 2.6. Assessment of Immunomodulators and Immunosuppressive Cytokines' Expression Profile

We quantified a group of key immunomodulators and tumor immunosuppressive cytokines. The *t*-test was utilized to compare the different expressions between the high-and low-risk score groups. The key immunomodulators include LAG-3, TIM-3, CTLA-4, IFN-*γ*, ICOS, ICAM-1, TIGIT, PD-1, PDL-1, NKG2A, and VISTA. Statistically, significance was considered when 2-sided *P* < 0.05.

## 3. Results

### 3.1. Overview of Pyroptosis-Related Genes in ccRCC

**Figure 2:**
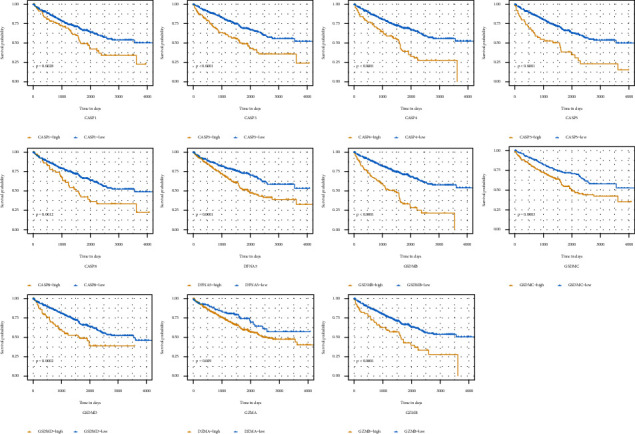
Survival analysis of pyroptosis-related genes in ccRCC. Kaplan–Meier curves for overall survival of 11 pyroptosis-related in the TCGA cohort.

**Figure 3:**
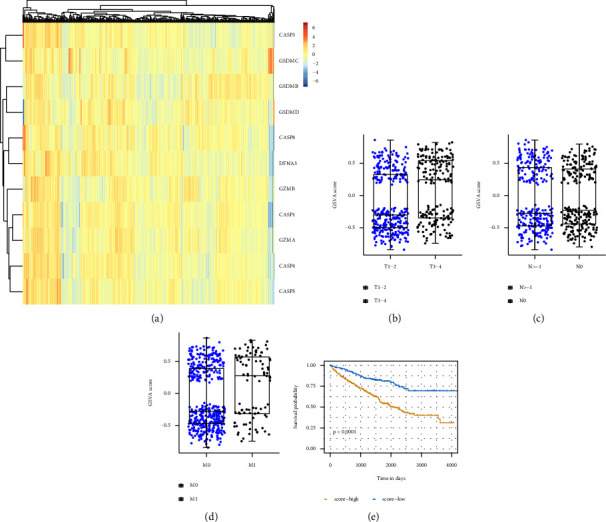
Significance of pyroptosis in ccRCC. (a) Heatmap of the pyroptosis-related genes in ccRCC cases. (b) Distribution of GSVA score of patients with different TNM tumor stages. (c) Distribution of GSVA score of patients with or without lymph node metastasis. (d) Distribution of GSVA score of patients with or without distant metastasis. (e) Kaplan–Meier curves for overall survival of GSVA score in TCGA cohort.

The selected 516 cancer cases from the TCGA database were pathologically diagnosed as ccRCC. The basic patient information and characteristics were shown in Table 1. Based on the Kaplan–Meier survival curves, the 11 pyroptosis-related genes were all significantly related to the overall survival (OS) outcome of the cancer cases with the log-rank test *P* < 0.05 (Figure 2). The distinct gene expression patterns of the 11pyroptosis-related genes in these cancer cases were presented in the heatmap (Figure 3(a)). Pyroptosis activity was calculated based on the pyroptosis signatures and GSVA. As shown in Figure 3(b), cancer cases with T staging III/IV, according to tumor node metastasis (TNM) classification, had higher GSVA scores than cancer cases with T staging I/II (*P* < 0.001).  Figure 3(c) exhibited that no significant difference was observed in GSVA scores when the cancer cases were grouped according to with or without regional lymph node metastasis. When compared to cancer cases without metastasis (M0), cancer cases with distant metastasis (M1) had significantly higher GSVA scores (Figure 3(d), *P* < 0.001). The cancer cases were divided into low-and high-GSVA score groups, based on the optimal cut-off value calculated by the survminer package in R. As shown in Figure 3(e), the cancer cases in the high-GSVA score group had a poorer prognosis, while cancer cased in low-GSVA score group had better OS (log-rank test *P* < 0.001).

### 3.2. Construction and Evaluation of the Pyroptosis Risk Model

Multivariate Cox regression analyses were used for the pyroptosis risk model establishment (Supplementary Table S1). Using the genes with a *P* value less than 0.1 within the supplementary table, the pyroptosis risk model was established. The risk score = 0.507955 ∗ Expression (CASP3) + 0.404610 ∗ Expression (CASP4) + 0.292399 ∗ Expression (GSDMB) + (−0.220535) ∗ Expression (GZMA).

**Figure 4:**
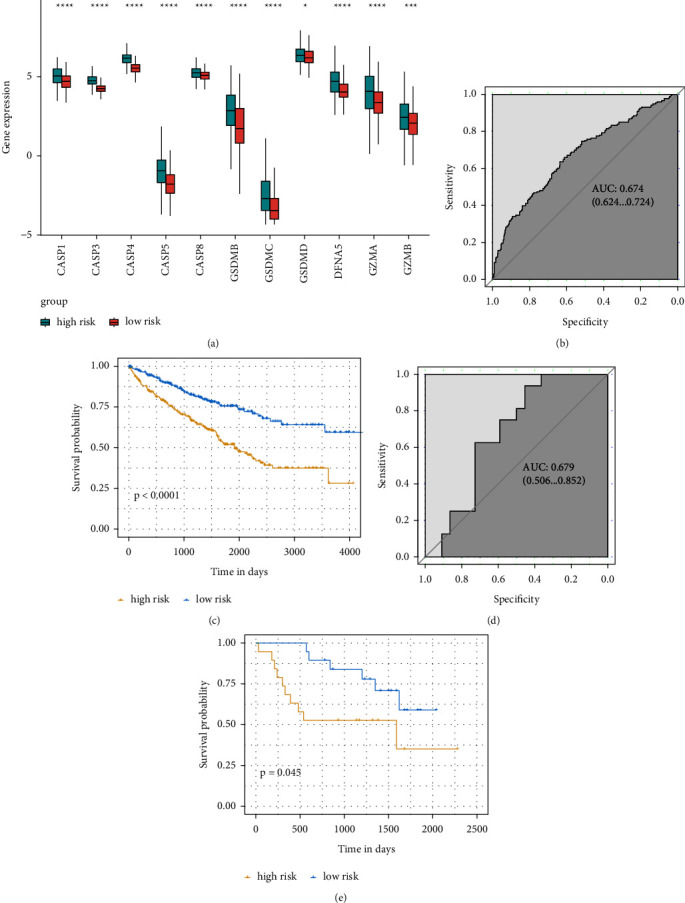
Pyroptosis risk model. (a) Distribution of genes in the pyroptosis risk model. (b)–(d) ROC analysis for the pyroptosis risk model in the TCGA cohort and GSE29609 cohort. (c)–(e) Kaplan–Meier curves for overall survival of risk score in the TCGA cohort and GSE29609 cohort. ROC, receiver operating characteristic. (^∗^: *P* ≤ 0.05; ^∗∗^: *P* ≤ 0.01; ^∗∗∗^: *P* ≤ 0.001; ^∗∗∗∗^: *P* ≤ 0.0001).

Following the median value of risk score (*P* < 0.05), all 516 ccRCC cases were divided into low- and high-risk groups. The whole of the 11 pyroptosis-related genes was upregulated in the high-risk score group (Figure 4(a)). ROC curves and Kaplan–Meier analysis were applied to assess the pyroptosis risk model. The risk model had an accuracy of 0.674 (95% CI: 0.624–0.724) in the TCGA cohort (Figure 4(b)). The cancer cases in high- risk score group had significantly poor OS (*P* < 0.001) (Figure 4(c)). To reveal the independent predictability of the risk model in predicting the prognosis of ccRCC, Cox proportional hazards regression analysis was performed and displayed in Table 2. The hazard ratio (HR) was 2.444 (95% CI 1.863–3.205) (*P* < 0.001).

Using the dataset GSE29609, external validation was performed. As shown in Figure 4(d), the area under the ROC curve (AUC) was 0.679 (95% CI: 0.506–0.852).  Figure 4(e) presented the same results that the cancer cases in high- risk score group had a significantly poor OS (*P*=0.045).

### 3.3. Functional Enrichment Analyses

**Figure 5:**
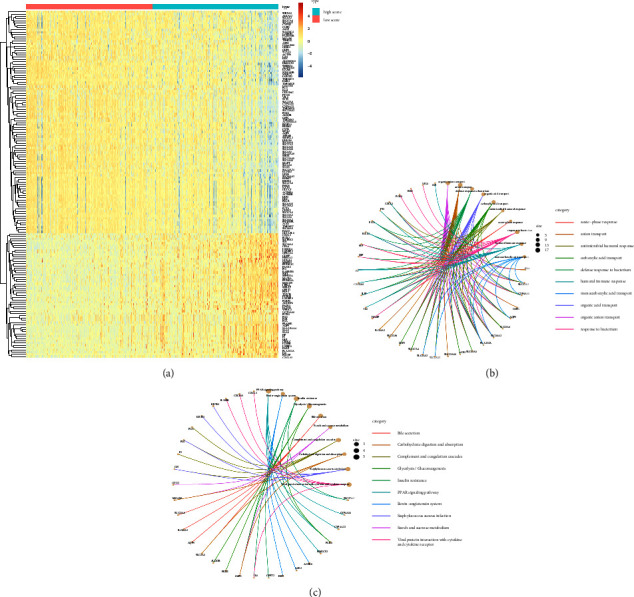
Analyses of DEGs. (a) Cases were divided into high-and low-risk score groups based on the median value. Heatmap of DEGs between high- and low-risk score groups. (b) Go enrichment analysis of DEGs. (c) KEGG pathway enrichment analysis of DEGs. DEGs, differentially expressed genes; GO, gene ontology; KEGG, Kyoto encyclopedia of genes and genomes.

DEGs between low-and high-risk score groups were identified. The profiles of DEGs expression for each group were exhibited in the heatmap (Figure 5(a)). GO and KEGG analyses were taken to appraise the biological involvement of the DEGs. As highlighted in Figure 5(b), the top GO terms comprised acute-phase response, carboxylic acid transport, humoral immune response, etc. Furthermore, KEGG analysis exposed that the DEGs were chiefly involved in carbohydrate digestion and absorption, complement and coagulation cascades, glycolysis/gluconeogenesis, PPAR signaling pathway, etc. (Figure 5(c)).

### 3.4. Immune Microenvironment of Low-and High-Risk Score Groups

**Figure 6:**
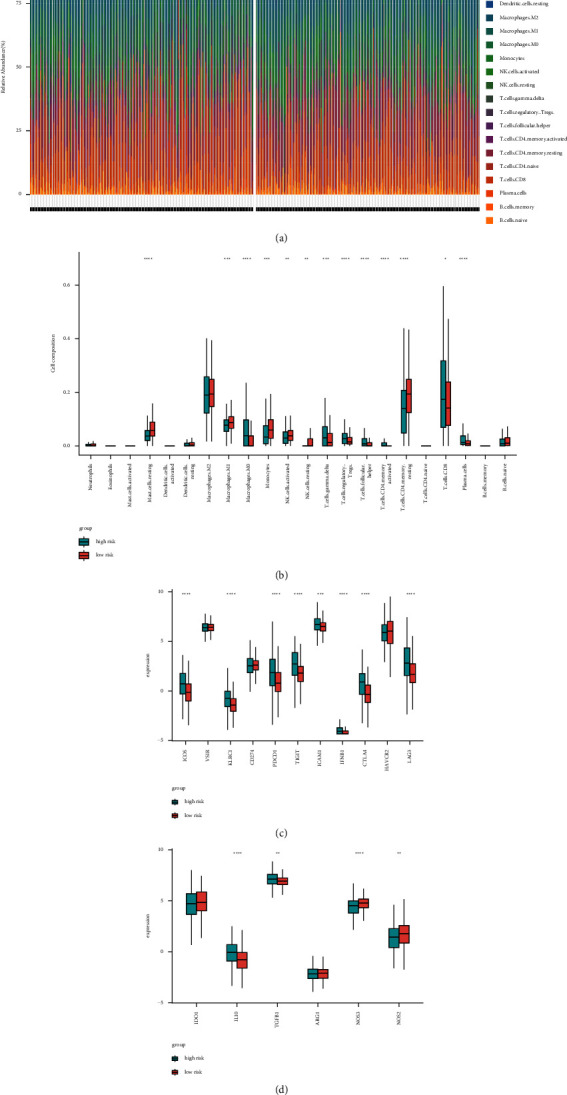
Immune landscapes between high-and low-risk score patients. (a) The abundance of immune infiltration in high-and low-risk score patients from TCGA cohort. (b) The proportions of different immune cells between high-and low-risk score groups in the TCGA cohort. (c)–(d) The proportions of immunomodulators and immunosuppressive cytokines between high-and low-risk score groups in the TCGA cohort. (^∗^: *P* ≤ 0.05; ^∗∗^: *P* ≤ 0.01; ^∗∗∗^: *P* ≤ 0.001; ^∗∗∗∗^: *P* ≤ 0.0001).

In consideration of the established pyroptosis risk model that could also reflect the immune microenvironment of ccRCC, the disparate immune cell fraction between low-and high-risk score groups was studied. The diverse immune cell fraction upshot of the 516 ccRCC cancer cases grouped into different risk score groups was depicted in Figure 6(a). In spite of the higher multiple effector immune cells (e.g. plasma cells, CD8+ T cells) in high-risk score groups, the immunosuppressive cells (e.g. regulatory T cells) were significantly higher in the same group (Figure 6(b)) (*P* < 0.05). This status may imply the immunosuppressive microenvironment in high-risk-score cancer cases.

In addition, we ferreted out the dissimilar expression of immunomodulators and immunosuppressive cytokines between low- and high-risk score groups. The expression of ICOS, KLRC1, PDCD1, TIGIT, ICAM1, IFNB1, CTLA4, and LAG3 were all significantly upregulated in the high-risk score group (Figure 6(c)) (*P* < 0.05). We discovered that several immunomodulators (e.g. TGF*β*1, IL10) were upregulated in the high-risk score group, while NOS2 and NOS3 were reduced (Figure 6(d)) (*P* < 0.05). Thusly, the cancer cases with a higher pyroptosis risk score may exist in an immunosuppressive microenvironment.

### 3.5. Single-Cell Transcriptomic Analysis of the Four Modeling Genes

**Figure 7:**
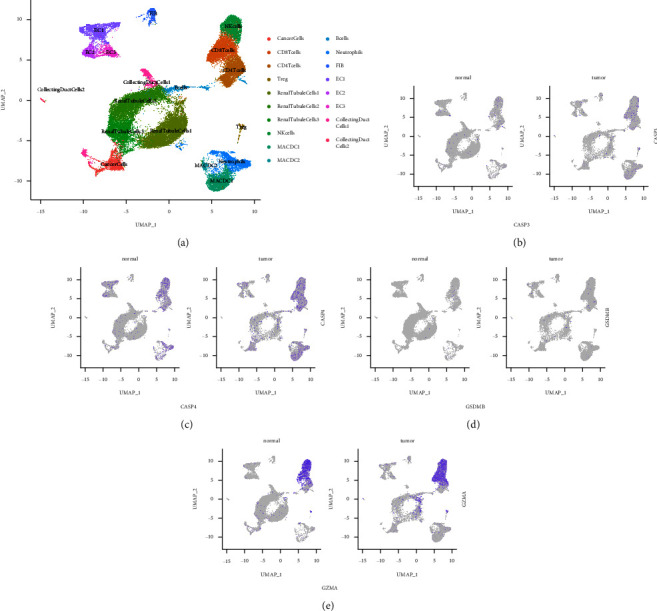
Pyroptosis-related genes observed at a single-cell level. (a) UMAP clustering of cells by differentially expressed markers. (b)–(e) Feature plots of CASP3, CASP4, GSDMB, and GZMA. UMAP, uniform manifold approximation, and projection.

In order to delve into the extra interrelation among the four modeling genes in ccRCC, single-cell transcriptomic data were exploited for further analysis. We identified 18 different cell clusters, including cancer cells, renal tubule cells 1, renal tubule cells 2, renal tubule cells 3, CD8+ T cells, CD4+ T cells, regulatory T cells (Treg cells), natural killer cells (NK cells), macrophages/dendritic cells (MACDC) 1, MACDC 2, B cells, neutrophils, fibroblasts (FIB), endothelial cells (EC) 1, EC 2, EC 3, collecting duct cells 1, and collecting duct cells 2 (Figure 7(a)). The different expression profiles of the four modeling genes in different types of cells were scrutinized. FeaturePlot revealed that the expression of the four modeling genes was higher in cancer samples than that in adjacent non-neoplastic samples (Figures 7(b)–7(e)).

## 4. Discussion

ccRCC is generally insensitive to radiotherapy and chemotherapy, the response rate of which to the targeted drugs and immunotherapy is lower, and at least partially resistant to damage of cell death-related signaling pathways. Pyroptosis was initially found in monocytes and macrophages and mediated primarily by the inflammasome–caspase-1 (CASP1) pathway [35]. Besides, caspase-3/4/5/8/11 (CASP3/4/5/8/11) was also involved in the regulation of pyroptosis [36–38]. The gasdermin superfamily protein is the main effector of pyroptosis. At present, the gasdermin superfamily members including GSDMA, GSDMB, GSMDC, GSDMD, and GSDME had been found [7]. In addition, when caspase-3 (CASP3) and granzyme B (GZMB) cleave gasdermin E (GSDME), granzyme A (GZMA) cleaves gasdermin B (GSDMB), cell apoptosis converts into pyroptosis pathway [30, 39, 40]. In the present study, we selected 11 pyroptosis-related genes including CASP1, CASP3, CASP4, CASP5, CASP8, GSDMB, GSDMC, GSDMD, GSDME, GZMA, and GZMB for subsequent analysis. Previous studies indicated that pyroptosis may be intimately related to tumorigenesis and tumor progression [35, 41]. It has been proved that the down-regulation of GSDMD could promote cell cycle arrest and activate ERK/STAT3/PI3K/AKT pathway in gastric cancer [42]. GSDMD could activate the EGFR/Akt pathway and promote the progression of the lung tumor [43]. In esophageal squamous cancer, GSDME overexpression indicated a better prognosis in patients [44]. In our study, the overexpression of 11 pyroptosis-related genes, individually, predicted poor overall survival of ccRCC. Pyroptosis activity of cases with T staging III/IV was higher than T staging I/II. Besides, ccRCC with distant metastasis had higher pyroptosis activity. In addition, higher pyroptosis activity means a poorer prognosis.

Due to the important role of pyroptosis-related genes in the occurrence, development, and prognosis of ccRCC, we constructed a pyroptosis risk model by multivariate COX regression analysis. The pyroptosis risk model, containing 4 genes namely CASP3, CASP4, GSDMB, and GZM, had the independent predictability of ccRCC prognosis in the TCGA cohort as well as the dataset GSE29609. In the functional enrichment analyses, the glycolysis signaling pathway was exhibited. It had been reported that glycolysis could play a key role in the process of proinflammatory activation during cell pyroptosis, in which interleukin (IL)-1*β* and IL-18 are released from plasma membranes. The metabolism of macrophages could switch from oxidative phosphorylation to glycolysis following proinflammatory activation [45]. Besides, the insulin resistance pathway was enriched in our study. Pyroptosis occurs not only in monocytes and dendritic cells but also in nonmacrophage cells [46–48]. It had been reported that adipose tissue also experiences pyroptosis [49, 50]. The intracellular concentration of LPS-inducing pyroptosis determined the adipocyte death size [51]. The adipocyte overexpansion induces a stress state, leading to obese adipocyte pyroptosis, which in turn recruits macrophages into adipose tissue and induces inflammation and insulin resistance in obese mice [50].

In addition, we explored the immune microenvironment of low pyroptosis and high pyroptosis risk score groups. In high pyroptosis risk score groups, the multiple effector immune cells such as plasma cells and CD8+ T cells were higher, possibly due to stimulation by inflammatory factors released during pyroptosis. However, the immunosuppressive cells such as regulatory T cells were also higher in the same groups, implying the immunosuppressive microenvironment induced by pyroptosis. In addition, we ferreted out the differentially expressed immune checkpoints. The expression of ICOS, KLRC1, PDCD1, TIGIT, ICAM1, IFNB1, CTLA4, and LAG3 were all significantly upregulated in the pyroptosis high-risk score group, indicating that pyroptosis could stimulate the activation of immune systems. In terms of immunomodulators, TGF*β*1 and IL10 were upregulated, while NOS2 and NOS3 were reduced in the pyroptosis high-risk score group. The discovered phenomenon of tumor immune microenvironment characterized the multiple impacts of pyroptosis. The pyroptosis risk score model may be useful in immunotherapy response grading and classification. The ccRCC with higher pyroptosis risk may benefit from the renovation of the immunosuppressive condition of the tumor microenvironment. These may provide ideas for preventing drug resistance and increasing the efficiency of immunotherapy.

However, there are still some defects in the study. We cannot avoid the potential for selection bias, since we drew the results based on the data downloaded from the TCGA database and GEO database. We cannot obtain additional detailed clinical information for further analysis. In this study, we exploited single-cell transcriptomic data to identify 18 different cell clusters, validating the extra interrelation among the four modeling genes in ccRCC. Further clinical trials and single-cell transcriptomic-based analysis should be performed to validate the pyroptosis risk model. Despite these defects listed above and the lack of further validation, the presented findings still proved the predicting ability of the pyroptosis risk model statistically and its potential application.

## 5. Conclusion

The study developed a pyroptosis-related risk model based on 4 identified pyroptosis-related genes. The conducted pyroptosis model could predict the prognosis of ccRCC and reflect the immune microenvironment, which may help in prognostic biomarker discovery in ccRCC patients and immune checkpoint inhibitor treatment in the future.

## Figures and Tables

**Table 1:** The basic clinical characteristics of the 516 cancer cases from the TCGA database.

	Subtype	No	Percent (%)
Age	≥60	277	53.68
	<60	239	46.32

Gender	Male	337	65.31
	Female	179	34.69

TNM staging	Stage I	254	48.64
	Stage II	55	10.66
	Stage III	122	23.64
	Stage IV	82	15.68

Survival status	Alive	343	66.47
	Dead	173	33.53

Total		516	100

**Table 2:** The univariate analysis and multivariate analysis of the risk score model.

Parameter	Univariate analysis			Multivariate analysis		
	HR	95% CI	*P*	HR	95% CI	*P*
Age	1.789	(1.316, 2.432)	< 0.001	1.835	(1.341, 2.510)	< 0.001
Gender	0.933	(0.685, 1.272)	0.662	0.959	(0.697, 1.319)	0.797
T staging	3.091	(2.280, 4.189)	< 0.001	1.868	(1.335, 2.615)	< 0.001
N staging	0.918	(0.681, 1.237)	0.575	0.809	(0.597, 1.096)	0.172
M staging	3.926	(2.884, 5.343)	< 0.001	2.565	(1.821, 3.613)	< 0.001
Risk score	2.964	(2.271, 3.867)	< 0.001	2.444	(1.863, 3.205)	<0.001

## Data Availability

The datasets used and/or analyzed during the current study are available from the corresponding author upon request.

## Supplementary Materials

Supplementary Materials In the Multivariate Cox regression analyses of the 11 pyroptosis-related genes, the *P* value of CASP3, CASP4, GSDMB, and GZMA is less than 0.1, and they were used for the establishment of the pyroptosis risk model.
