# Supplementary material for: Pyroptosis-Related Gene Signature Predicts the Prognosis of ccRCC Using TCGA and Single-Cell RNA Seq Database
Source: J Healthc Eng. 2022 Oct 30;2022:8224618. doi: 10.1155/2022/8224618 (PMC9637477; doi:10.1155/2022/8224618)
Supplement: Supplementary Materials — In the Multivariate Cox regression analyses of the 11 pyroptosis-related genes, the P value of CASP3, CASP4, GSDMB, and GZMA is less than 0.1, and they were used for the establishment of the pyroptosis risk model. [file 8224618.f1.docx]

Table S1 Multivariate Cox regression analysis of the 11 pyroptosis related genes

|  | HR | 95%CI | *P* |
| --- | --- | --- | --- |
| CASP1 | 0.8582 | 0.5824 1.2647 | 0.4397 |
| CASP3 | 1.6619 | 1.0571 2.6126 | 0.0278 |
| CASP4 | 1.4987 | 0.9896 2.2698 | 0.0561 |
| CASP5 | 1.1313 | 0.9229 1.3868 | 0.2351 |
| CASP8 | 0.9967 | 0.6423 1.5467 | 0.9883 |
| GSDMB | 1.3396 | 1.1738 1.5289 | 0.0000144 |
| GSDMC | 0.9331 | 0.8136 1.0702 | 0.3222 |
| GSDMD | 1.1260 | 0.8337 1.5209 | 0.4389 |
| GSDME | 1.0960 | 0.8806 1.3642 | 0.4115 |
| GZMA | 0.8021 | 0.6540 0.9837 | 0.0342 |
| GZMB | 1.1156 | 0.9286 1.3403 | 0.2427 |
